# Can we use serum gamma-glutamyl transferase levels to predict early mortality in stroke?

**DOI:** 10.12669/pjms.303.4456

**Published:** 2014

**Authors:** Emine Akinci, Nurettin Özgür Doğan, Haluk Gümüş, Nazire belgin Akilli

**Affiliations:** 1 Emine Akinci, Keçiören Training and Research Hospital, Emergency Department, Ankara, Turkey.; 2 Nurettin Özgür Doğan, Kocaeli University Medicine Faculty, Emergency Department, Kocaeli, Turkey.; 3 Haluk Gümüş, KonyaTraining and Research Hospital, Neurology Department, Konya, Turkey.; 4 Nazire belgin Akilli, KonyaTraining and Research Hospital, Emergency Department, Konya, Turkey.

**Keywords:** Stroke, GGT, Mortality

## Abstract

**Objective:** Serum gamma-glutamyl transferase (GGT) is a marker for alcohol consumption and hepatobiliary diseases. There are reports on the prognostic role of GGT in coronary artery diseases and stroke. The aim of our study was to identify the potential differences in GGT levels in different types of stroke, and to evaluate the correlation between GGT and 30-day mortality.

**Method:** Patients diagnosed with stroke in emergency department between 01.01.2010 and 30.12.2012 was included in the study. Imaging techniques were used to distinguish between hemorrhagic and ischemic stroke. Ischemic strokes were further classified as either atherosclerotic/lacunar or embolic. Parameters including age, gender, vital signs (systolic and diastolic blood pressure), comorbid diseases (HT, DM, CAD, smoking and alcohol consumption), used medications, previous history of stroke, NIHSS score at the time of admission to emergency department, laboratory parameters (glucose, white blood cell count, hemoglobin, platelet, total cholesterol, creatinine) and duration of hospitalization were recorded. Death records were obtained from patients’ medical records.

**Results:** One thousand eighty six patients were included in the study. GGT levels were not significantly different between ischemic and hemorrhagic strokes (p=0.435). On the other hand, GGT levels in embolic strokes were significantly higher compared to atherosclerotic/lacunar strokes (p=0.001). GGT levels [median 24.50 (16.00-43.00)] in Intensive Care Unit patients were significantly higher compared to GGT level [22.00 (15.00-34.25)] in admitted to service beds patients (p=0.015). Median GGT level of deceased patients was 24.00 (16.00-41.25) and median GGT level of alive patients was 22.00 (15.00-35.00). GGT level of deceased patients was significantly higher compared to GGT levels of alive patients (p=0.048).

**Conclusion:** There was no difference in GGT levels between ischemic and hemorrhagic strokes; however, GGT levels in embolic strokes were significantly higher compared to atherosclerotic/lacunar strokes. High GGT levels are correlated with early mortality in stroke. We believe that GGT may be used as a predictor of mortality in future studies.

## INTRODUCTION

Serum gamma glutamyltransferase (GGT) is an affordable, highly-sensitive and reliable laboratory test which is frequently used as a hepatobiliary dysfunction and alcohol abuse indicator.^1^ It is known that in half of the people who use alcohol regularly, 40-60g daily, there is an increase in serum levels of GGT.^2^ Recently, GGT has been shown to have an active role in oxidative and inflammatory mechanisms as well as atherosclerotic pathogenesis, and thus it can be used as a biomarker to determine risk for cardiovascular diseases.^3^^-^^6^ Risk factors for coronary artery diseases and stroke have also been indicated.^7^^,^^8^ In addition, GGT has a prognostic role in CVD and stroke.^9^ The aim of our study was to identify the differences in GGT levels in different types of stroke, and to evaluate the relationship between GGT and 30-day mortality.

## METHODS

This retrospective study was approved by the local ethics committee. Patients who were admitted to Emergency Department of Konya Training and Research Hospital in first 24 hours diagnosed with stroke and admitted in neurology and intensive care units (ICU) between the dates January 1, 2010 and December 30, 2012 were included in this study. Data was obtained from patients’ medical records. Neurologic examinations of the patients were evaluated using National Institutes of Health Score Scale (NIHSS) score. Patients with scores 1-4 were considered to have mild neurological deficits, patients with scores higher than 5 were considered to have severe neurological deficits.^9^

**Table-I:** Selection of study participants

*Characteristics of patients that excluded from the study*	*N:1874*
Patients for whom laboratory findings could not be reached	511
Patients for whom alcohol consumption could not be reached	308
Patients who had no CT	46
Patients in which diffusion MR was not present	370
Patient who could not be fully evaluated in terms of cardiologic status	287
Patients who had arrest in emergency services and from whom blood samples were taken after the treatment	31
Patients with chronic renal failure, cirrhosis, and high enzyme levels	233
Presence of history of any cancer	88

**Table-II:** Demographical and laboratory data of the patients

	*Ischemic Stroke*				
	*Atherosclerotic*	*Embolic*	*p value*	*Hemorrhagic stroke*	*p value*
Age	75.00 (64.50-82.00)	74.00(64.00-81.00)	0.589	72.00 (59.50-80.00)	0.003
Gender (male)	359 (53%)	102 (40.6%)	<0.001	78 (48.4%)	0.783
Hemoglobin g/dL	13.80 (12.50-15.20)	13.60 (12.50-14.80)	0.358	13.55 (12.68-14.93)	0.768
WBC 10^3/µL	8.40 (6.70-10.90)	8.26 (6.60-10.74)	0.754	9.52 (6.51-11.27)	<0.001
Platelet 10^3/µL	225 (181.25-273.00)	228 (180-273)	0.824	222 (186.75-280.00)	0.524
Glucose mg/dL	123 (99-172)	119 (96.00-161.50)	0.182	121 (95.00-152.50)	<0.001
Creatinine mg/dL	0.80 (0.70-1.10)	0.80 (0.70-1.04)	0.007	0.77 (0.69-0.97)	0.101
GGT U/L	21.5 (13-25)	25 (17-40)	0.001	23.00 (15.75-36.50)	0.435
Total cholesterol mg/dL	184 (156.00-212.75)	183.5 (150.00-212.75)	0.170	167.5 (149.25-214.75)	0.978
Systolic blood pressure	160 (145-180)	155 (140.00-174.25)	0.119	186.50 (169.75-210.75)	<0.001
Diastolic blood pressure	85 (77-90)	85 (78-90)	0.780	100.00 (89.75-108.50)	<0.001
NIHSS	4 (2-9)	4 (1-10)	0.031	5.50 (2.50-10.75)	<0.001
EF	60 (55-64)	60 (51.5-62.0)	0.171	60 (55.00-63.50)	0.652
Hospitalization period	6 (4-11)	7 (4-11)	0.380	9.50 (7.00-15.25)	0.186
Number of patients at ICU	207 (30.6)	85 (33.9%)	0.026	109 (67.7%)	<0.001
Mortality	211 (31.2%)	87 (34.7%)	0.082	107 (66.5%)	<0.001

**Table-III:** Patient history and previously used medications

	*Ischemic Stroke*				
	*Atherosclerotic*	*Embolic*	*p value*	*Hemorrhagic stroke*	*p value*
HT	490 (72.4%)	181 (72.1%)	0.870	128 (79.5%)	0.057
AF	50 (7.4%)	140 (55.8%)	<0.001	11 (6.8%)	<0.001
DM	214 (31.7%)	75 (29.9%)	0.855	39 (24.2%)	0.076
Coronary Artery Disease	260 (38.4%)	103 (41.0%)	0.128	41 (25.5%)	0.001
Smoking	286 (42.2%)	103 (41.0%)	0.030	49 (30.4%)	0.006
Anti-aggregant therapy	290 (42.8%)	113 (45.0%)	0.215	54 (33.8%)	0.022
Anti HT therapy	426 (62.9%)	159 (63.3%)	0.030	91 (56.5%)	0.123
Antilipid therapy	193 (28.5%)	68 (27.1%)	0.060	30 (18.6%)	0.011
Previous history of stroke	162 (23.9%)	79 (31.5%)	0.061	32 (19.9%)	0.107

The distinction between hemorrhagic and ischemic stroke was made according to noncontrast computed tomography (CT) and diffusion MR examinations. Gradient echo sequence was used in some of the patients, so it was not included in the study. The etiology of the patients who had ischemic stroke was completed according to the Toast classification. Lacunar and atherosclerotic infarcts were evaluated together. Patients who were of unknown etiology or due to other reasons were excluded from the study. Patients with ischemic stroke were divided into two groups: embolic and atherosclerotic/lacunar. The distinction between atherosclerotic/lacunar and embolic was made according to the presence of atrial fibrillation (AF), the characteristics of the lesion in CT and diffusion MR, the presence of thrombus in the echocardiography and Doppler ultrasonography, valvular heart disease, and a history of the use of anticoagulants (warfarin) and the history of the patients (such as a recent attack of MT, the presence of cardiomyopathy, valvular disease, chronic sinoatrial dysfunction, interatrial septal anomaly).

Considering the medications used and previous medical records, hypertension (HT), diabetes mellitus (DM) and coronary artery disease (CAD) were found in patients. Death records were obtained from patients’ medical records and medical records.

***Exclusion criteria were:*** (1) giving blood after intravenous fluid replacement or treatment by any means (resuscitation etc.) in emergency department, (2) presence of known chronic liver or kidney diseases, (3) presence of thyroid dysfunctions, (4) presence of active infections, (5) history of neoplasia, (6) previous high aspartate transaminase and alanine transaminase enzymes, (7) alcohol consumption (The patients with intermediate and severe alcohol use (2)), (8) presence of transient ischemic attack (TIA), (9) admission to the hospital 24 hours after the first symptom, (10) absence of laboratory results, (11) general cardiac evaluation (history, physical examination, electrocardiography, transthoracic and/or transasephageal echocradiography) and (12) absence of Doppler ultrasonography (13) Those patients for which CT and diffusion MR could not be performed (14) high levels of liver enzymes or functional impairment due to drug use.

The age, gender, vital signs (systolic, diastolic blood pressure), comorbid diseases (HT, DM, CAD, history of smoking and alcohol), medications used, previous history of stroke, NIHSS scores at the time of evaluation in the emergency department, laboratory parameters (glucose, white blood cell (WBC), hemoglobin, platelet, total cholesterol, creatine), duration of hospitalization and mortality of the patients were recorded on the study forms.

Hematology parameters were assessed using Sysmex XE-2100 (Kobe, Japan), Biochemistry parameters were assessed using Abbott C-16000 autoanalyzer (Abbott Laboratories, Abbott Park, Illinois, USA) in the study. 

Statistical Analysis: SPSS 15.0 statistical software was used for data analysis. Kolmogorov-Smirnov test was used to evaluate the coherence of the data with normal distribution. Student t-test was used in pairwise group analysis of continuous variables which were normally distributed, and the data were represented as mean +/- standard deviation. Mann-Whitney U test was used in pairwise analysis of continuous and ordinal variables which were not normally distributed, and the data were represented as median (quarters of 25-75%). Categorical data was compared using Pearson chi-square test and was represented as numbers-percentages. Spearman method was used for correlation analysis. P values <0.05 were considered as statistically significant.

## RESULTS

Medical records of 2960 patients were examined and 1086 eligible patients (i.e. according to the criteria used) were included in the study. Characteristics of patients that excluded from the study were shown Table-I.

Among all participants, 161 (14.8%) patients had hemorrhagic and 925 (85.2%) had ischemic stroke. 674 (62.1%) of ischemic stroke patients had stroke in the atherosclerotic/lacunar base, 251 (23.1%) of ischemic stroke patients had stroke in the embolic base. Basic demographic features of the patients and some laboratory values are presented in Table-II. Accordingly, GGT levels did not differ between ischemic and hemorrhagic stroke (p=0.435) whereas they were significantly high in embolic strokes compared with atherosclerotic/lacunar strokes (p=0.001). GGT levels were not significantly different between males and females (p=0.143).

The patients were evaluated according to their background features such as DM, HT, CAD and previously used medications (Table-III).

Regarding GGT levels of the patients, GGT levels [median 24.50 (16.00-43.00)] in Intensive Care Unit patients were significantly higher compared to GGT levels [22.00 (15.00-34.25)] in service patients (p=0.015). Similarly, median GGT level of deceased patients was 24.00 (16.00-41.25) and median GGT level of alive patients was 22.00 (15.00-35.00). GGT level of dead patients was significantly higher compared to GGT levels of alive patients (p=0.048).

Regarding NIHSS scores of the patients, median level for service patients was 3 (2-4); median level for Intensive Care Unit patients was 14 (11-17) (p<0.001). According to GGT levels for NIHSS scores, median GGT level in acute stroke was 24 (16-40) and median GGT level in mild strokes was 22 (15-35); however, there was no significant difference (p=0.062).

GGT levels in DM (p=0.174), HT (p=0.687), CAD (p=0.799) patients were not found significantly different. In addition, GGT levels between smoker and non-smoker were not found significantly different (p=0.110). Antiaggregant treatment (p=0.745), antihypertensive treatment (p=0.584) and antilipid treatment (p=0.528) did not change GGT levels significantly.

Median GGT level of patients with atrial fibrillation was 25.00 (17.00-40.25); median GGT level of patients without atrial fibrillation was 22 (15-36); GGT levels of patients with atrial fibrillation were significantly high compared with patients without atrial fibrillation (p=0.008).

There was a significantly weak negative correlation between GGT levels and age (r=0.079, p=0.011), whereas there was a weak positive correlation between GGT and glucose levels (r=0.156, p<0.001). The correlation between GGT levels and parameters including duration of hospitalization, ejection fraction and NIHSS score were not significant.

## DISCUSSION

According to our study, high GGT levels were associated with 30-day mortality in stroke patients. GGT levels in deceased patients were significantly higher compared to alive patients. Moreover, GGT levels of Intensive Care Unit patients were significantly higher compared to service patients.^10^

GGT and cardiovascular events were examined in several prospective studies.^11^^-^^13^ These studies indicate that basal GGT level has a positive correlation with cardiovascular cases and mortality risk. Although the relationship between GGT and atherosclerosis is well-known, the exact mechanisms underlying the relation between GGT and atherosclerosis remain unclear. There are some proposed mechanisms. The first mechanism may be that GGT is related to many of the atherosclerotic risk factors. Previous studies showed that serum GGT levels were found to be related to hypertension, metabolic syndrome and DM.^14^^-^^16^ However, according to our results, GGT is not associated with total cholesterol, DM and HT. Similarly, GGT levels did not change in patients who receive antiplatelet, antihypertensive and antilipid treatment.

GGT is expressed not only in liver and kidney, but also cerebrovascular endothelium, pericytes and other cell types.^17^ Therefore, GGT can be used as a biochemical marker which is able to cross the blood-brain barrier.^18^ GGT is released by atherosclerotic plaques in damaged cerebral endothelial cells; this finding may explain the predictive value of GGT in fatal cerebrovascular cases. Korantzopoulos et al. unveiled that GGT was higher in patients with first-time ischemic stroke than control group patients without ischemic stroke.^8^ Bolt et al. also showed in Eurostroke Project that increased GGT levels were correlated with hemorrhagic stroke.^7^ Comparing with ischemic cerebrovascular events, intracerebral hemorrhages have been demonstrated to causing more mortality (bir kaynak eklenebilir). Nevertheless, GGT levels were not different in each stroke subtypes. Mortality might be also depending on several mechanical factors such as mass effect or other vascular problems in hemorrhagic strokes. In our study, we divided ischemic stroke into two main groups: Atherosclerotic/lacunar and embolic. Unlike the aforementioned study, we found higher GGT levels in embolic stroke. In particular, GGT levels in patients with atrial fibrillation were higher compared to the patients without atrial fibrillation. In previous studies, there was an independent positive correlation between serum GGT levels and atrial fibrillation.^19^ There was also thrombocyte hyperactivity in paroxysmal and chronical atrial fibrillation.^20^^-^^22^ Thrombocytes are considered to have the GGT4 isoenzyme. Thus, increased thrombocyte activity and continuous thrombocyte breakdown may lead to increased serum GGT activity.^23^^,^^24^ GGT determined mortality from all causes, MI, stroke and Cardiac death. An epidemiological investigation (Vorarlberg Health Monitoring and Promotion Program) including 163,944 participants showed that strong evidence of positive associations between high GGT and mortality from chronic forms of coronary heart disease, congestive heart failure and ischemic or hemorrhagic stroke in both men and women.^9^ Emdin et al. showed that serum GGT activity had a prognostic value for cardiac death and nonfatal MI in patients with documented atherosclerotic CAD and earlier MI.^14^ Ulus et al. assessed GGT activity and major adverse cardiac event (mortality from cardiac causes, recurrent hospitalization with ACS and nonfatal recurrent MI diagnosis, to need for coronary revascularization during in Coronary Care Unit (CCU)) patients due to acute coronary syndrome for 1-6 months period.^25^ They showed that increased GGT activity, even in normal limits, is an independent prognostic factor for adverse cardiac event development in relatively high risk ACS patients. According to our results, high GGT levels are correlated with early mortality in stroke independently of alcohol consumption. 

## CONCLUSION

GGT levels were not significantly different between ischemic and hemorrhagic stroke, while GGT levels were statistically higher in embolic stroke compared to atherosclerotic/lacunar stroke. High GGT levels are associated with early mortality in stroke. We suppose that GGT can be used as a predictor of mortality in future. 

## Notes

***Limitations of the study:*** This study is retrospective and study data were obtained from patients’ medical records. We evaluated the level of GGT at the time of admission to emergency services and we did not follow up GGT in certain intervals. We were not able to reach body height and weight of the patients. We did not analyze the presence of metabolic syndrome. Furthermore, patients were evaluated for only 30-day mortality, not for long-term mortality.
